# Study on the Interfacial Functionary Mechanism of Rare-Earth-Solution-Modified Bamboo-Fiber-Reinforced Resin Matrix Composites

**DOI:** 10.3390/ma11071190

**Published:** 2018-07-11

**Authors:** Kaikui Zheng, Chenghui Gao, Fushan He, Youxi Lin, Ming Liu, Jiao Lin

**Affiliations:** 1 School of Mechanical Engineering and Automation, Fuzhou University, Fuzhou 350116, China; kuikui@fzu.edu.cn (K.Z.); hfshan@fzu.edu.cn (F.H.); lyx@fzu.edu.cn (Y.L.); mingliu@fzu.edu.cn (M.L.); n150220045@fzu.edu.cn (J.L.); 2 Mechanical and Electrical Engineering Practice Center, Fuzhou University, Fuzhou 350116, China

**Keywords:** rear earth, modification, bamboo fiber, resin, interface, mechanism

## Abstract

In this work, a new and effective treatment on bamboo fiber (BF) is presented, and its effect on the interfacial bonding properties of the BF/resin matrix was studied. The interfacial functionary mechanism of rare earth solution (RES) modification to improve the interfacial bonding properties between BF and the resin matrix was analyzed. The hardness and elastic modulus of the interfacial zone between BF and the resin matrix were measured using nanoindentation. Fourier-transform infrared spectroscopy (FT-IR) was used to analyze the change in the surface functional group of BF in the modification process. The surface chemical composition of BF before and after the modification was characterized by X-ray photoelectron spectroscopy (XPS). The results show that the RES modification significantly increases the hardness and elastic modulus of BF and its interfacial zone with the resin matrix. The hydroxyl concentration on the surface of RES-treated BF decreases, which reduces the hydrophilicity of BF. Rare earth ions react with oxygen in the hydroxyl group at the C2 position in the glucosylic ring of cellulose. The RES-modified BF bonds with the resin matrix to form a rare earth complex, which significantly enhances the interfacial adhesion between BF and the resin matrix.

## 1. Introduction

Natural-plant-fiber-reinforced composites are among the fastest-growing products of all composites in recent years. As a type of environmentally-friendly material, natural-fiber-reinforced resin matrix composites have become new materials in the twenty-first century [1,2,3,4,5,6]. Natural fiber has a high specific strength and specific modulus, which makes the new, reinforced resin-based brake material have biodegradability, small pair damage, and good wear resistance at low temperatures [7,8,9,10,11]. In the study of natural plant fiber/resin composites, the strengthening effect of the components on the matrix depends on the fiber structure and the interface between fiber and resin. Due to the presence of many polar hydroxyl groups on the surface of natural fiber, natural fiber shows notably strong polarity and hydrophilicity, which results in its poor interfacial compatibility with the hydrophobic resin matrix [12,13,14,15]. The interfacial adhesion between the untreated fibers and the resin matrix is notably poor. The fiber is easily detached from the resin matrix during the friction process, which decreases the properties of the composites [16,17]. Many researchers used different physical or chemical modification methods to try and improve the interface adhesion between natural fibers and the resin matrix [18,19,20,21,22,23,24,25,26,27,28,29]. For example, Bledzki and Gassan put together an excellent review on the most-used chemical or physical methods for surface modification of natural fibers [18]. The results of Rodríguez and Francucci’s study [19] showed that the composites manufactured with alkali-treated fibers and coated with polyhydroxybutyrate exhibited the best performance in terms of mechanical properties. Tzounis et al. [20] performed a carbon nanotube modification on natural fibers. They found that the excellent interfacial adhesion of carbon nanotube-coated jute fiber with the natural rubber matrix contributed to a significant improvement on tensile strength and tensile modulus. Orue et al. [21] studied the effects of different chemical treatments on sisal fiber bundles’ tensile properties and the tensile properties of composites. They found that composites based on alkali and NaOH+silane treated sisal fibers showed better mechanical properties than untreated ones. Fiore et al. [22] performed commercial sodium bicarbonate surface treatment on sisal fiber. They found that treated fibers showed notable improvements in mechanical properties, compared to the raw one in which the T-24 h fibers showed improvements up to about 138.5% and 63.2% of the tensile strength and modulus, respectively. Erdoğan et al. [23] studied the effects of various surface treatment processes on waste jute fibers. The results showed that fluorocarbon treatment with alkali pretreatment can be more useful than other surface treatments in short fiber-reinforced composite. 

In previous studies [30,31,32], alkali treatment, steam blasting treatment, polyvinyl chloride (PVC) coating treatment, and other methods have been used to modify the surface of BF, which can effectively improve the interfacial bonding properties between BF and the resin matrix. The study of interfacial modification has become the key point of improving the performance of natural-plant-fiber-reinforced resin matrix composites. Rare earth elements are gradually introduced into the preparation of composites because of their excellent physical and chemical characteristics which improve the heat resistance, mechanical properties, interfacial properties, and so forth, of many materials [33,34,35]. In a previous work [36], the effects of RES on the mechanical and tribological properties of BF-reinforced brake composites were studied. We found that the RES modification can significantly improve the mechanical and tribological properties of the brake composites. The main highlights of this work, compared to the previous paper, are to investigate the effects of RES modification on the interfacial bonding properties between BF and the resin matrix from the microcosmic structure of the interface, and to study the mechanism of the interaction between RES-modified BF and the resin matrix, which can be used to explain the RES modification to improve the macro-mechanical and tribological properties of the composites. For this purpose, the mechanical properties of the composites were characterized for tensile strength, flexural strength, fracture toughness, and impact toughness. The hardness and elastic modulus of the interfacial zone between BF and resin was tested using nanoindentation and nanomechanical microscopy, in order to characterize the interfacial bonding properties of the BF/resin matrix composites. Scanning electron microscopy (SEM), Fourier-transform infrared spectroscope (FT-IR), and X-ray photoelectron spectroscopy (XPS) were used to analyze the changes in morphology, surface functional group, and chemical composition in BF because of the RES treatment. This interfacial functionary mechanism with interfacial adhesion properties serves as a guide to the development of the surface modification of natural plant fibers, for high performance and biodegradable brake composites, and advanced natural-fiber-reinforced resin matrix composite applications.

## 2. Materials and Methods 

### 2.1. Formulation and Designation of Composites

**Figure 1:**
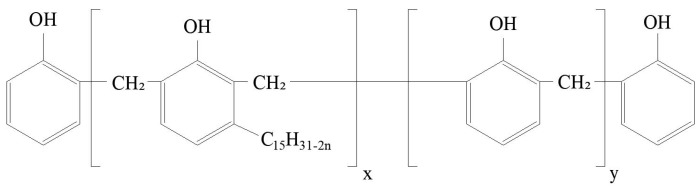
The general chemical structure of used phenolic resin.

BF was supplied by Fujian Zheng He, Bamboo Spinning Co., Ltd. (Longyan, China). Table 1 outlines the details of the BF, which were provided by the supplier. Rare earth lanthanum chloride powder was used for the surface modification of BF, which was procured from Shanghai Macklin Biochemical Technology Co., Ltd. (Shanghai, China). Cashew nut-shell-liquid-modified phenolic resin was supplied by Sumitomo (Tokyo, Japan). Table 2 outlines its details, which were provided by the supplier. The general chemical structure of used phenolic resin is shown in Figure 1.

### 2.2. Fabrication of Composites

In a previous work [36], the effects of RES modification with a different concentration and processing time on the mechanical and tribological properties of bamboo-fiber-reinforced resin matrix brake composites were investigated. We found that the optimization of the mechanical property of RES-modified composites was obtained, as the process parameters were: RES concentration of 10%, processing time of 30 min. The RES modifier was prepared by using absolute ethanol as the solvent and lanthanum chloride (LaCl_3_) as the solute, and the solution, with a mass fraction of 10%, was prepared. The BF was immersed in RES and soaked for 30 min; afterwards it was removed and placed into a drying box (Model JF980S, Jilin Electrical and Mechanical Equipment Co., Ltd., Changchun, China) at 100 °C for 2 h for drying. The RES modification was compared with alkali treatment to show the advantages of RES modification on the mechanical properties of composites. In a previous study [37], the optimization of the mechanical property of alkali-treated composites was obtained when the BF was immersed in 5% alkali solution and soaked for 48 h. The alkali-treated BF was prepared using the same process. The formulation of the composites for tensile, flexural, fracture toughness, and impact toughness testing are presented in Table 3.

To better study the effects of RES modification on the interfacial bonding properties of BF and resin, the composites for interfacial bonding properties testing were prepared with only 20% BF and 80% phenolic resin. The ingredients were mixed in a mixer (Model JF810S, Jilin Electrical and Mechanical Equipment Co., Ltd., Changchun, China) to ensure macroscopic homogeneity. The mixing was performed for 15 min. Then, the mixture was placed in a four-column hydraulic machine (Model Y32-63, Ruian Huada Machinery Co., Ltd., Ruian, China). The mold cavity was filled with approximately 60 g of the mixture and heat-cured in a compression molding machine under a pressure of 10 MPa for 10 min, at a curing temperature of 160 °C. Five intermittent breathings were provided to allow the volatiles to be expelled during the initiation of curing. Then, the composites were post-cured in an oven (Model JF980S, Jilin Electrical and Mechanical Equipment Co., Ltd., Changchun, China) at 160 °C for 12 h. The post-cured composites were surface-grinded, polished, and used for further characterization.

### 2.3. Measurements

#### 2.3.1. Mechanical Characterization

The mechanical properties of the composites were characterized for tensile strength, flexural strength, fracture toughness, and impact toughness. The tensile strength and flexural strength of the composites were measured using a microcomputer-controlled electronic universal testing machine (Model CMT-5105, New Sice Materials Testing Co., Ltd., Shenzhen, China), according to the ISO 527-4 [38] and ISO 14125 [39] standards, respectively. The flexural strength, $\sigma_{f}$, is given by:

(1) $$\sigma_{f}=\frac{3P\cdot l}{2b\cdot h^{2}}$$

where *P* is the fracture load, *l* is the span length, *b* is the width of the sample, and *h* is the thickness of the sample. The flexural strain, ε, can be calculated by:

(2) $$\varepsilon =\frac{6S\cdot h}{l^{2}}$$

where *S* is the deflection at the middle point of the span. The impact strength of the composites was measured using a simply-supported beam pendulum impact tester (Model XJJ-5, Jinan Fangyuan Test Instrument Co., Ltd., Jinan, China), according to the GB/T 1451-2005 standard [40]. The fracture toughness of the composites was measured using a microcomputer-controlled electronic universal testing machine (Model Instron 1185, Instron, Norwood, MA, USA), according to the GB 4161-1984 standard [41]. The fracture toughness of the composites was measured by using the three-point bending method. The fracture toughness, *K*_1*c*_, is computed by:

(3) $$K_{1c}=\frac{P_{c}S}{BW^{\frac{1}{2}}}f(\frac{a}{W})$$

where *P_c_* is the fracture load, *S* is the span length, *B* is the thickness of the sample, *W* is the width of the sample, *a* is the crack length, and $f(\frac{a}{W})$ is the geometric factor. $f(\frac{a}{W})$ can be given by:

(4) $$f(\frac{a}{W})=\frac{3{\left(\frac{a}{W}\right)}^{\frac{1}{2}}[1.99-\frac{a}{W}(1-\frac{a}{W})(2.15-3.93\frac{a}{W}+2.7\frac{a^{2}}{W^{2}})]}{2(1+2\frac{a}{W}){\left(1-\frac{a}{W}\right)}^{\frac{3}{2}}}$$

Each case was measured 5 times, after which the results were averaged. All mechanical tests were performed at room temperature.

#### 2.3.2. Morphological Characterization

Morphological studies of untreated and RES-treated BF were conducted with SEM using a Quanta 250 machine (FEI, Hillsboro, OR, USA). The RES-treated BF was immersed in the 10-percent-RES and soaked for 30 min, and then it was put into a drying box (Model JF980S, Jilin Electrical and Mechanical Equipment Co., Ltd., Changchun, China) at 100 °C for 2 h for morphological characterization. The fracture morphology of samples after impact testing was analyzed by SEM (FEI, Hillsboro, OR, USA). All samples were coated with a thin layer of gold using a sputtering coater (Model S150RTL, Cowaq Company, Hertfordshire, UK).

#### 2.3.3. Characterization of the Interfacial Bonding Properties

**Figure 2:**
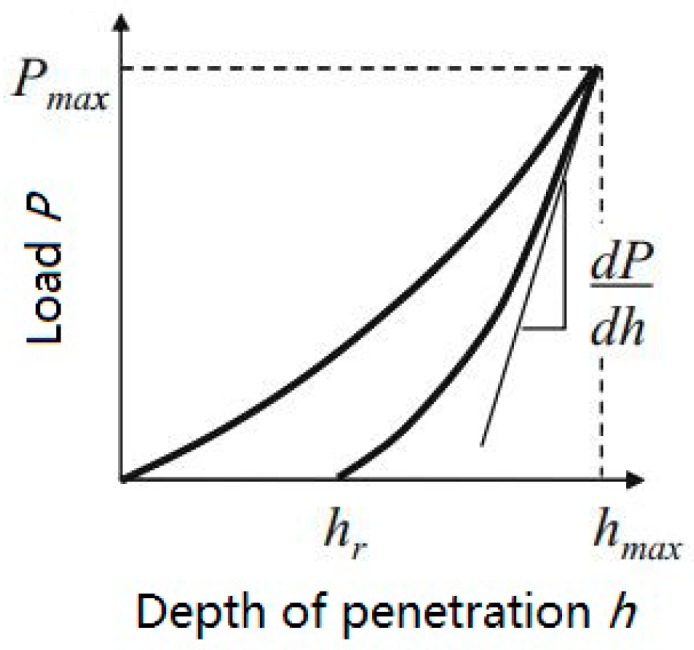
Load-displacement curves for the elastic-plastic solid.

The nanoindentation tester (Model NHT2, Anton Parr, Graz, Austria) was used to test the hardness and elastic modulus of the interfacial zone between the BF and the resin matrix. The nanoindentation technique has become widely available and used extensively to study interfacial mechanisms and properties (e.g., hardness [42], bonding strength [43], interfacial properties [44]). The two most commonly measured properties when using a nanoindentation test are elastic modulus and hardness. In this study, the contact between the diamond indenter and the resin matrix composites is the elastic-plastic contact between the ball and the plane. The load-displacement curves for the elastic-plastic solid are shown in Figure 2.

The indentation modulus is usually determined from the slope of the unloading curve at maximum load. The indentation modulus (here expressed as E*), as a function of *dP*/*dh* and the area of contact, can be expressed as:

(5) $$E^{*}=\frac{1}{2}\frac{\sqrt{\pi }}{A}\frac{dP}{dh}$$

The indentation hardness, *H*, is calculated from the indentation load divided by the projected contact area, and is given by:

*H* = *P*/*A* (6)

where *A* is the projected area of contact.

#### 2.3.4. FT-IR Spectroscopy

To analyze the change in the surface functional group of BF during the modification process, the FT-IR spectra of untreated BF and RES-treated BF were recorded on a FT-IR spectrometer (Model Nicolet-5700, Nicolet Instrument Corporation, Madison, WI, USA) for the infrared representation in the wave number range of 4000~400 cm^−1^ with a resolution of 4 cm^−1^. The BF was made into a powder, mixed with KBr, and made into thin sheets.

#### 2.3.5. XPS Analysis

The surface chemical composition of BF before and after the modification was characterized using an ESCALAB 250 XPS (Thermo VG Scientific, Waltham, MA, USA). With Al K Alpha as the source of XPS excitation, the electron-binding energy of C1s is 284.6 eV was the internal standard. First, a wide-spectrum scan was performed on the untreated and RES-treated BF. The pass energy was 100 eV, and the energy step was 1 eV. Then, a high-precision narrow spectrum scan was performed for C1s, O1s and La3d. The pass energy was 30.0 eV, and the energy step was 0.05 eV.

## 3. Results and Discussions

### 3.1. Characterization of Mechanical Properties

**Figure 3:**
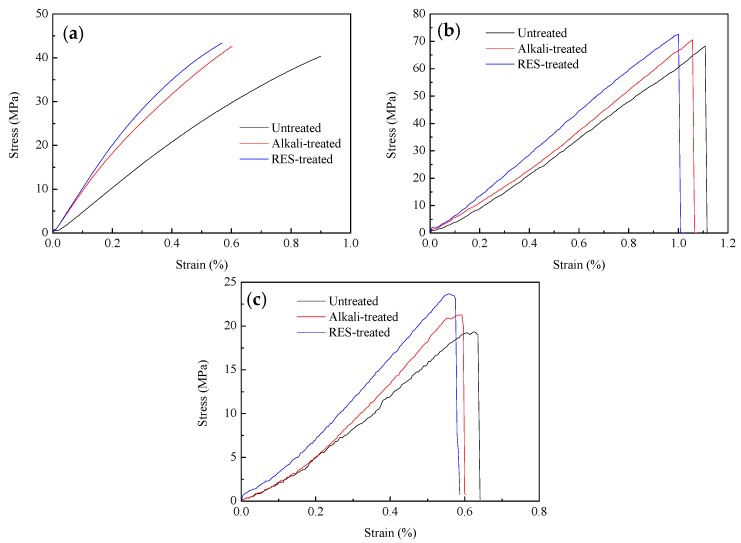
The stress–strain curves of composites: (**a**) tensile test; (**b**) flexural test; (**c**) fracture toughness test.

The test results of the tensile, flexural, and fracture toughness of the composites are shown in Figure 3 and Table 4. From Figure 3, it can be seen that the RES-treated composites are superior to the alkali-treated and untreated composites in most properties, such as tensile strength, elastic modulus, flexural strength, and fracture toughness, while the untreated composites are superior in strain at break. As seen from the data in Table 4, the properties of tensile strength, flexural strength, fracture toughness, and impact strength of the composites increased due to the RES modification and alkali treatment. Compared with untreated composites, the tensile strength, flexural strength, fracture toughness, and impact strength of the RES-treated composites increased by 8%, 6%, 22% and 13%, while the tensile strength, flexural strength, fracture toughness, and impact strength of the composites treated by alkali increased by 6%, 3%, 8% and 8%, respectively. It can be seen that RES modification on the mechanical properties of composites is better than that of alkali treatment, especially considering the improvement in fracture toughness.

### 3.2. Morphology Analysis

#### 3.2.1. Surface Morphology of BF

**Figure 4:**
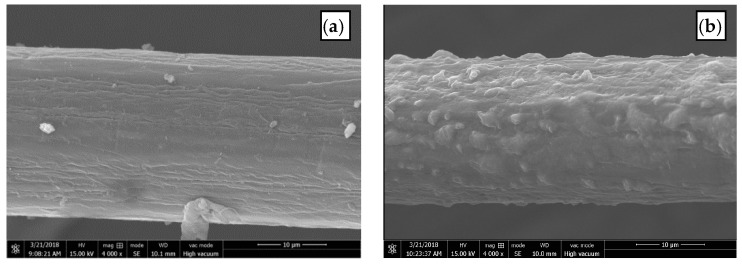
SEM images of the surface morphology of BF: (**a**) untreated; (**b**) RES-treated.

The SEM images of untreated and RES-treated BF are shown in Figure 4, which display the surface morphology of the BF before and after treatment. The surface of the untreated BF (Figure 4a) is smooth, and there are low molecular impurities, such as pectin, lignin, and hemicellulose, on the surface of the fiber. The surface of the RES-modified BF (Figure 4b) is relatively rough, and the surface area increased, improving the mechanical meshing force between the fiber and the matrix.

#### 3.2.2. Fracture Morphology of Composites

**Figure 5:**
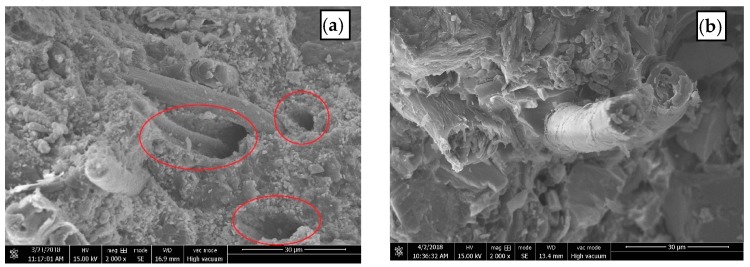
SEM images of the fracture morphology of samples after impact testing: (**a**) untreated; (**b**) RES-treated.

The SEM images of the fracture morphology of impact specimens are shown in Figure 5. From Figure 5a, it can be seen that some BF from the untreated composites are pulled out of the pits from the resin matrix (mark as the red circles), which shows the phenomenon of interfacial debonding and fiber pullout, indicating that the bond strength between the BF and the resin matrix is weak. From Figure 5b, it can be seen that the fracture of the BF can be observed on the cross-section of RES-treated composites, and the fracture of the fiber and the port of the composite is flat, which shows the good interfacial bonding between the BF and the resin matrix.

### 3.3. Characterization of Interfacial Bonding Properties

**Figure 6:**
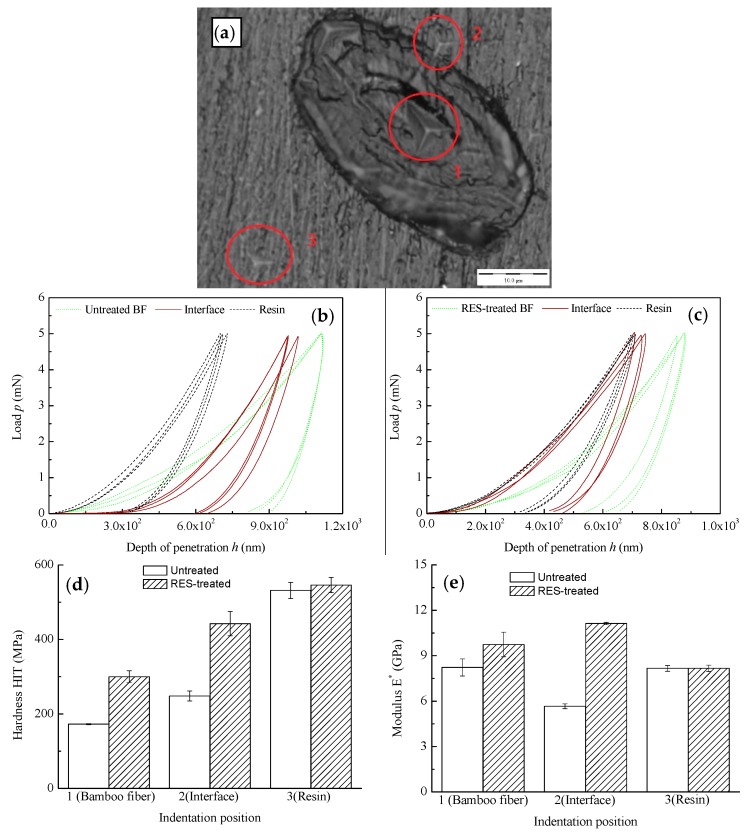
Indentation hardness and elastic modulus of each test point in the interface zone between fiber and resin: (**a**) the selection of indentation test position; (**b**) load-displacement curves of untreated composites; (**c**) load-displacement curves of RES-treated composites; (**d**) indentation hardness of composites; (**e**) indentation elastic modulus of composites.

The hardness and elastic modulus of the interfacial zone between BF and resin was tested using the nanoindenter. The interfacial zone of BF-reinforced resin composites was found using nanoscale microscopy. The hardness and elastic modulus of the BF, BF/resin interface, and resin matrix were measured by the nanoindenter. The selected indentation test positions are shown in Figure 6a. The indentation test points for the BF surface, BF/resin interface, and resin matrix are marked as 1, 2, and 3, respectively. Each indentation test point was subjected to 3 repeated tests, and the average value was taken as the hardness and elastic modulus of each point. The load-displacement curves of each test point are shown in Figure 6b,c, and the hardness and elastic modulus of each point are shown in Figure 6d,e.

Figure 6b,d shows that the hardness of the surface of the BF and BF/resin interface obviously increased after the RES modification, which indicates that the RES-modified BF can improve the hardness of the surface of the BF and BF/resin interface. The reason for this is because of the introduction of rare earth elements on the surface of the BF after RES modification. The rare earth compound has a large hardness [45,46], meaning that the hardness of the fiber surface and the interface between BF and resin can be improved.

Figure 6c,e shows that the elastic modulus of BF and the interface between BF and resin obviously increased after the RES modification, which indicates that the RES modification can improve the elastic modulus of BF and the BF/resin interface. The interface between untreated BF and resin has a significantly lower elastic modulus than the BF and resin matrix, which indicates the poor interfacial bonding property between the hydrophilic BF and the hydrophobic resin matrix. The interface between RES-treated BF and resin has the highest elastic modulus (11.13 GPa), followed by BF (9.74 GPa), and the resin matrix has the lowest elastic modulus (8.16 GPa). The interfacial adhesion between the fiber and the resin matrix has been significantly improved after the modification. The interface between BF and resin has a greater elastic modulus than the reinforced fiber and resin matrix. We can speculate that the modified BF and the resin matrix have a chemical combination in the composite process, which greatly improves the interfacial bonding properties between BF and resin.

### 3.4. Infrared Spectroscopic Analysis

**Figure 7:**
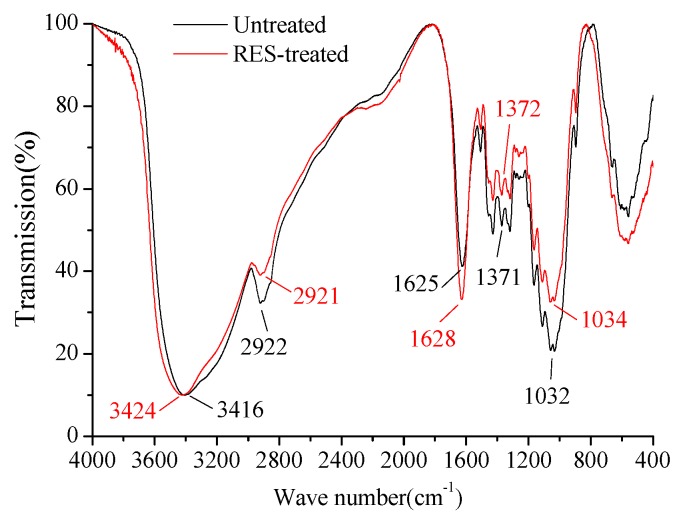
FT-IR spectra of BF.

FT-IR can help identify the functional groups in the BF to analyze the change in the surface functional group of BF in the modification process. The FT-IR spectrum of untreated and RES-treated BF is shown in Figure 7. Similar to most natural fibers, the peak in the region of 3200–3600 cm^−1^ corresponds to the axial stretching of the hydroxyl group (–OH) [47,48], which is notably common in natural fibers and attributed to hydrocarbon constituents. The characteristic peaks in the regions of 3000~2900 cm^−1^, 1800~1600 cm^−1^, 1400~1300 cm^−1^ and 1100~1000 cm^−1^ are designated as the peaks of C–H [49], C=O [50], C–H [24,51] and C–O [52], respectively. The peak assignments of the absorption bands that correspond to various groups are summarized in Table 5.

Figure 7 shows that the O–H stretching vibration frequency and bandwidth of the BF have changed after the modification. The peak of the O–H stretching vibration of untreated BF is located at 3416 cm^−1^, and the bandwidth is wide. The peak of the O–H stretching vibration of RES-treated BF is located at 3424 cm^−1^, and the bandwidth is narrow. The O–H stretching vibration frequency is commonly used to detect the strength of hydrogen bonds. A lower vibration frequency and a wider band indicate a stronger hydrogen bond [53]. The O–H stretching vibration frequency increased and the bandwidth narrowed after the RES modification, which indicates that the hydrogen bonds among large molecular chains of cellulose were weakened, the hydrophilicity of the BF decreased, and the interfacial compatibility between the BF and the resin matrix was improved.

### 3.5. X-ray Photoelectron Spectroscopy Analysis

#### 3.5.1. Wide-Scan Photoelectron Spectra of BF

**Figure 8:**
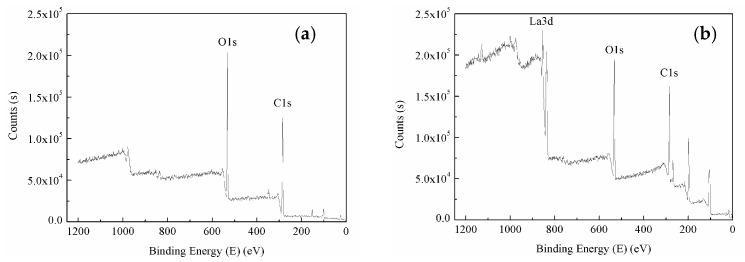
Wide-scan photoelectron spectra of BF: (**a**) untreated; (**b**) RES-treated.

The XPS spectrum of untreated and RES-treated BF is shown in Figure 8.

**Figure 9:**
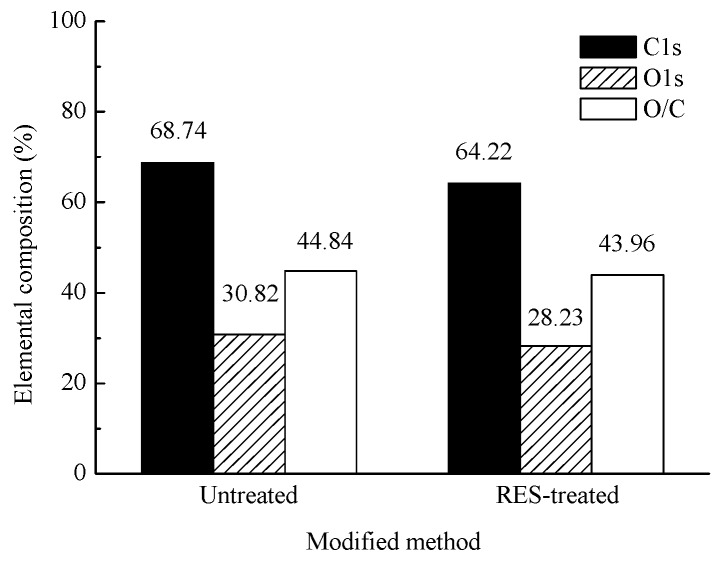
Surface elementary composition of untreated and RES-treated BF.

Figure 8 shows the characteristic peaks of C1s and O1s on the surface of the untreated and RES-treated BF, which indicates that C and O are the main elements of natural BF, conforming to the composition of the cellulose molecular chain of BF. The surface of the RES-modified BF exhibits the characteristic peaks of La3d and C1s. The characteristic peaks of C1s and O1s changed in the spectrogram. The relative content of C and O on the surface of the modified BF decreased because of the introduction of lanthanum on the surface of the BF. Each glucose radical ring in the natural cellulose chain contains three hydroxyl groups at the 2-, 3- and 6-positions of carbon atoms, which include two secondary alcohol hydroxyl groups and one primary alcohol hydroxyl group [54]. The presence of these hydroxyl groups directly affects the chemical properties of cellulose. These hydroxyl groups constitute the strong hydrogen bonds among and within large molecular chains of natural cellulose, which make the natural fibers exhibit strong polarity and hydrophilicity and are difficult to be compatible with the hydrophobic polymer matrix. The surface hydroxyl concentration of BF can be characterized by the relative content of O and O/C to a certain extent. The content and changes to the surface elements of the untreated and RES-treated BF are shown in Figure 9.

In Figure 9, the relative content of C and O on the BF’s surface decreases after the RES modification, and the O/C rate decreases also. BF modification by RES can reduce the hydroxyl concentration of the BF’s surface, reduce the polarity and hydrophilicity of the natural BF, and improve the interfacial bonding property between the fiber and the resin matrix.

#### 3.5.2. Narrow-Scan Photoelectron Spectra of BF

**Figure 10:**
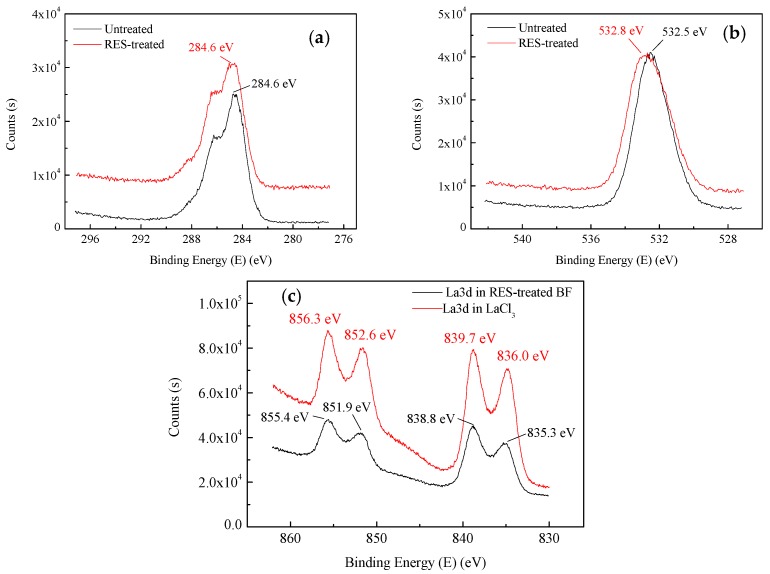
Narrow-scan photoelectron spectra of elements: (**a**) XPS spectra of C1s; (**b**) XPS spectra of O1s; (**c**) XPS spectra of La3d.

The XPS results of C1s and O1s on the surface of untreated and RES-treated BF are shown in Figure 10a,b. The binding energy at 284.6 eV is the characteristic peak of C1s on the surface of untreated and RES-treated BF. The binding energy at 532.5 eV is the characteristic peak of O1s on the surface of untreated BF, while the binding energy at 532.8 eV is the characteristic peak of O1s on the surface of RES-treated BF. The XPS results of La3d on the surface of RES-treated BF and La3d in LaCl_3_ (solvent in RES) are shown in Figure 10c. The absorption peaks of La3d on the surface of RES-treated BF were found at 855.4 eV, 851.9 eV, 838.8 eV and 835.3 eV, while the absorption peaks of La3d in LaCl_3_ were found at 856.3 eV, 852.6 eV, 839.7 eV and 836.0 eV.

**Figure 11:**
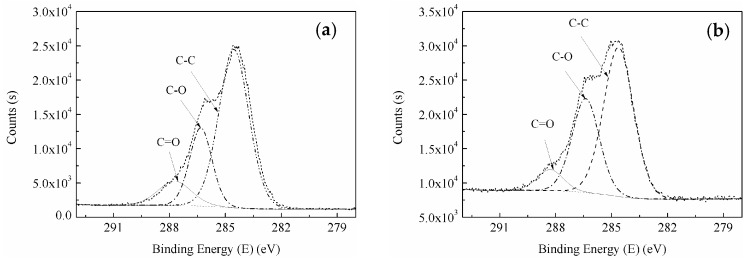
Curve fit of C1’s photoelectron peak of the BF: (**a**) untreated; (**b**) RES-treated.

From Figure 10a,b, we observe that the C1s’ peak of BF before and after the modification did not change, whereas the binding energy of O1s of RES-treated BF increased by 0.3 eV compared to the untreated BF. Compared with the absorption peaks of La3d in LaCl_3_ (Figure 10c), the La3d absorption peaks on the surface of RES-treated BF moved towards low binding energy. Because of the larger coordination number of rare earth elements [55], La^3+^ is bonded with O on the surface of BF during the modification, and the La atom receives a negative charge from the O coordination atom. Therefore, the binding energy of La3d can be reduced, and the O1s binding energy can be increased. To further analyze the changes of the surface functional groups during the modification process, the C1s spectrum peaks of the XPS spectrum of untreated and RES-treated BF were fitted, as shown in Figure 11.

**Figure 12:**
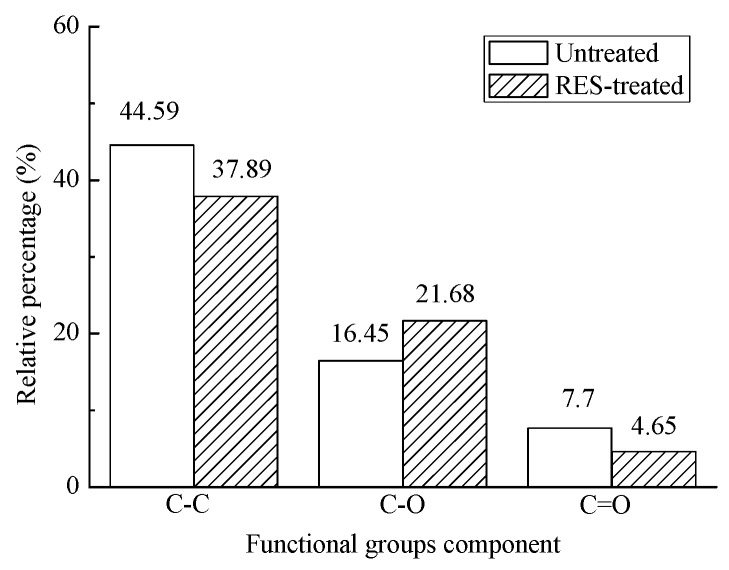
Relative percentage of the groups from the C1s peak curve fitting of BF.

The C1s spectrum peak of BF can be divided into three main peaks for fitting. The binding energies of 284.6 eV, 286.3 eV, and 287.7 eV correspond to the C–C, C–O, and C=O groups, respectively. Figure 11 shows that the area between the single peak and the baseline of each functional group in the C1s spectrum peaks have changed, which implies that the relative content of the functional group of the BF changes after modification. The relative content of surface groups before and after the BF modification is shown in Figure 12.

In Figure 12, the relative content of C–C and C=O groups on the surface of the BF decreased after the RES modification, whereas the C–O group increased. The O content did not increase after the modification (Figure 9), which indicates that the C–O group on the BF’s surface mainly increases because of the transformation of the C=O group.

### 3.6. Mechanism of RES Modification

**Figure 13:**
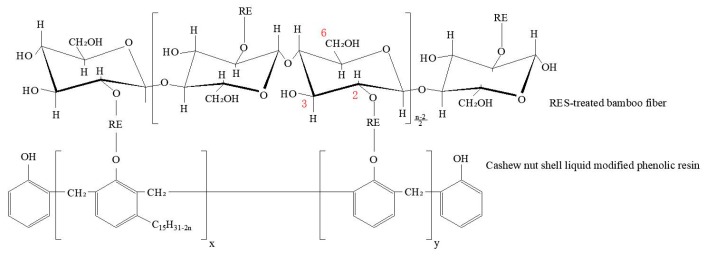
Mechanism of the improvement of interfacial adhesion between BF and the resin matrix by the RES modification.

Rare earth has a unique 4f electronic structure, abundant energy level transitions, and excellent interfacial properties. Lanthanum is the most active element in rare earth elements and can be chemically bonded with most non-metallic elements. The size of the rare earth ions is large, and the complexes have higher coordination numbers. Rare earth ions contain unfilled 4f electrons, which cannot completely shield the nuclear charge, so they have a lively chemical property. During the modification process, La^3+^ coordinates with the oxidation of the hydroxyl of BF and chemically adsorbs onto the surface of BF. There are three hydroxyl groups on each glucose base ring at 2-, 3- and 6-positions of carbon atoms, where the hydroxyl at the 2-position of carbon atoms (C2) shows the strongest acidity. Therefore, the coordination reaction with La^3+^ should be the hydroxyl on C2, as shown in Figure 13. Because of the high coordination number of rare earth elements, when the RES-treated BF is combined with the phenolic resin matrix, La^3+^ can still be coordinated with the oxygen groups on the molecular chain of the phenolic resin.

## 4. Conclusions

This study aims to use its findings as a guide to the development of this new and effective surface modification of natural plant fibers for advanced green composite applications. The results obtained are useful for understanding the interfacial functionary mechanism of the RES modification in order to improve the interfacial bonding properties between BF and the resin matrix. The results are summarized as follows:

1. The RES modification on the mechanical properties (tensile strength, flexural strength, fracture toughness, and impact strength) of composites is better than that of alkali treatment, especially in regard to the improvement of fracture toughness. The surface of BF modified by RES becomes rough and the surface area increases, which improves the mechanical meshing force between the fiber and the matrix.
2. The RES modification can improve the hardness and elastic modulus of the BF and the interface between BF and the resin matrix; in particular, the interfacial zone has a larger elastic modulus than BF and the resin matrix, which indicates the formation of chemical bonds between the modified BF and the resin matrix during the composite process.
3. The O–H stretching vibration frequency increased and the bandwidth narrowed after the RES modification, which indicates that the hydrogen bonds among large molecular chains of cellulose were weakened, the hydrophilicity of BF decreased, and the interfacial compatibility between BF and the resin matrix was improved.
4. The RES modification caused the relative contents of C and O on the BF’s surface and the O/C rate to decrease, indicating that modification of the BF by use of RES can reduce the hydroxyl concentration of the BF’s surface, reduce the polarity and hydrophilicity of the natural BF, and improve interfacial bonding properties between the fiber and the resin matrix.
5. During the modification process, La^3+^ coordinates with the oxidation of the hydroxyl of BF and chemically adsorbs onto the surface of BF. La^3+^ can also be coordinated with the oxygen groups on the molecular chain of phenolic resin, which forms a stable structure of rare earth complexes.

## Figures and Tables

**Table 1:** Details of Bamboo fiber (BF) used in composites.

Physical Properties		Mechanical Properties		Chemical Composition (%)			
Density (g·cm^−3^)	Diameter (µm)	Tensile Strength (MPa)	Tensile Modulus (GPa)	Cellulose	Hemicellulose	Lignin	Others
1.34	10–25	750	35	58.5	18	22	1.5

**Table 2:** The technical index of resin used in composites.

Sieve Analysis Test 160-Mesh Sieve (%)	Curing Time 150 °C (s)	Flow Distance 125 °C (mm)	Viscosity (mPa·s)	Molecular Weight	PH	Free Phenol (%)
≤5	50~100	40~80	3~4	600~700	>7	≤5

**Table 3:** Formulation of composites (mass fraction, %).

Bamboo Fiber	Resin	Alumina	Barium Sulfate	Copper	Rubber Powder	Graphite
7	25	25	20	15	5	3

**Table 4:** Mechanical properties of the composites.

Properties	Untreated Composites		Alkali-Treated Composites		RES-Treated Composites	
	Average	Standard Deviation	Average	Standard Deviation	Average	Standard Deviation
Tensile strength (MPa)	40.24	1.41	42.85	0.66	43.56	0.49
Flexural strength (MPa)	68.05	0.72	70.22	0.66	72.33	0.65
Fracture toughness ($\mathrm{MPa}\sqrt{m}$)	1.46	0.01	1.57	0.09	1.78	0.07
Impact strength (kJ/m^2^)	6.33	0.29	6.85	0.08	7.13	0.06

**Table 5:** Assignment of the wave number in the IR spectra of BF before and after the modification.

Wave Number (cm^−1^)		Functional Group Assignment
Untreated BF	RES-Treated BF	
3416	3424	O–H, stretching vibration
2922	2921	C–H, stretching vibration
1625	1628	C=O, stretching vibration
1371	1372	C–H, deformation
1032	1034	C–O, stretching vibration
